# Analysis of Required Investigations of Work-Related Musculoskeletal Disorders in Spain

**DOI:** 10.3390/ijerph16101682

**Published:** 2019-05-14

**Authors:** Jesús Antonio Carrillo-Castrillo, Ventura Pérez-Mira, María del Carmen Pardo-Ferreira, Juan Carlos Rubio-Romero

**Affiliations:** 1 School of Industrial Engineering, Camino de los Descubrimientos sn, Universidad de Sevilla, 41092 Seville, Spain; vperez4@us.es; 2 School of Industrial Engineering, C/ Dr. Ortiz Ramos s/n, Campus de Teatinos, Universidad de Málaga, 29071 Málaga, Spain; carmenpf@uma.es (M.d.C.P.-F.); juro@uma.es (J.C.R.-R.)

**Keywords:** cause investigation, musculoskeletal disorders, occupational ergonomics, preventive measures, risk factors

## Abstract

Musculoskeletal disorders (MSD) are the most common source of occupational health problems in Western countries. In Spain, musculoskeletal disorders (MSDs) can be reported either as accidents or occupational diseases. When reported as an occupational disease, a full diagnosis is performed, as the compensation system needs the approval of the social security authority and a mandatory investigation has to be performed. Although many methods are available for investigating the causes of occupational accidents, occupational diseases have not been analysed with the same depth, and there is a lack of investigation methods. This paper aims to analyse the role of 43 occupational investigations of causes of musculoskeletal diseases in the prevention cycle. This study is based on the occupational investigations performed by workplaces’ occupational health and safety specialists when musculoskeletal diseases are reported. The analysis of the data involves descriptive statistics and the Φ coefficient. Based on administrative data, 68 workplaces employing 15,260 workers were surveyed and 41 workplaces with 13,201 workers submitted valid questionnaires to be analysed. The most frequent cause of reported musculoskeletal disease, in terms of primary risk, is repetitive movement. The only proposed measure with a significant association to the exposure by repetitive movements is job rotation (alternating workers between tasks within a job or between activities as a means to vary different levels of exposure). The investigation of occupational diseases has been useful in most of the cases for proposing preventive measures. Most of the workplaces surveyed have performed investigations and adopted preventive measures, but the managers of some workplaces were not aware of any disease notification regarding their workers when surveyed. More research is needed to provide tools for this important task.

## 1. Introduction

Musculoskeletal disorders (MSDs) continue to be a major source of disability and lost work time [1]. The importance of MSDs in the European Union has been analysed by the European Agency for Safety and Health at Work [2], showing that MSDs are by far the most significant occupational health problem. At the same time, the cost of MSDs is considerable to the social security systems of Spain and most Western countries [3,4]. These facts justify the need for a better understanding of the causes of MSDs and possible interventions to prevent them, including the need for a holistic approach [5].

In order to satisfy this need, it is important that accidents and occupational diseases are reported. However, not all accidents and occupational diseases are reported at work. According to Punnet and Wegman [6], whether occupational diseases are reported at work is likely affected by differences in pain threshold, cultural influences, psychosocial factors, employer receptivity, job insecurity and labour relations, among other factors. Despite these factors, it is necessary that this information be reported and adequately investigated to improve the prevention of MSDs. 

### 1.1. Reporting of Occupational Musculoskeletal Disorders in Spain

In Spain, MSDs can be reported either as accidents or as occupational diseases. If the injury is related to movements whereby the injured person’s physical exertion exceeds what is normal, it is classified as a musculoskeletal accident. If the damage has resulted from the long-term influence of working conditions, it is reported not as an accident, but as an occupational disease. According to this reporting criterion we will use the word ‘disease’ only for those musculoskeletal disorders reported as occupational diseases, and ‘accident’ for those musculoskeletal disorders reported as accidents. 

However, the reporting system in Spain relies on subjective medical criteria and does not always include a full diagnosis, so it can misclassify injuries as diseases and diseases as injuries. Nonetheless, once the disorder is reported, it is possible to reclassify it correctly if a full diagnosis is performed, as the compensation system needs the approval of the Social Security authority [7]. The system for official notification of occupational diseases in Spain is called CEPROSS (Sistema de Comunicación de Enfermedades Profesionales en la Seguridad Social—Social Security Notification System for Occupational Diseases) [8]. 

In CEPROSS, cases reported are coded. The codification of occupational diseases reported is approved by the Spanish government [9] with six groups of diseases. Among these groups, group 2 is used for the codification of diseases caused by physical agents. Within group 2, there are several subgroups (from 2A to 2I). Subgroup 2A is for hypoacousis and deafness related to noise exposure and subgroup 2I is for diseases caused by ionising radiation, so they are not included in this research. The rest of the subgroups (2B to 2G) are related to forced or awkward postures and repetitive movements, so they are included in this research (see Table 1 for the subgroup descriptions) as they are likely musculoskeletal disorders. These codes for MSDs in Spain adapted the EU groups of the European Commission Recommendation of 19 September 2003 concerning the European schedule of occupational diseases.

Accordingly, the number of occupational diseases related to musculoskeletal disorders included in those subgroups 2B to 2G reported in Andalusia in 2010 is presented in Table 1, with both codes according to the European Commission Recommendation of 19 September 2003 concerning the European schedule of occupational diseases and Spanish classification of diseases. 

Regarding Andalusia, there were 2868 workers in 2010 (data from Andalusian Labour Force Survey available [10]. Thus incidence rate of musculoskeletal diseases reported in 2010 is 0.15 per thousand workers. Within the workplaces with MSD reported included in this study, the number of workers in 2010 is 28,306. Thus, the incidence rate of musculoskeletal diseases reported for these workplaces in 2010 is 15.79 per thousand workers. Therefore, these workplaces present a high incidence rate. It must be considered that unemployment rates in Andalusia were increasing since 2007 with 12.75% to 2010 with 27.77%.

### 1.2. Risks and Causes of Musculoskeletal Disorders

Epidemiological analysis has identified collectives of workers at risk and risk factors of MSDs [11]. A more recent review confirmed that risk factors with at least reasonable evidence of a causal relationship for the development of work-related musculoskeletal disorders include not only heavy physical work but also smoking, high body mass index, high psychosocial work demands and the presence of co-morbidities [12]. In the same review, the most commonly reported biomechanical risk factors with at least reasonable evidence for causing MDSs include excessive repetition, awkward postures and heavy lifting. Psychosocial work stressors also show a remarkable importance as antecedents of musculoskeletal problems [13].

In an extensive review of the literature by the National Institute of Occupational Safety and Health, evidence was found for the causal relationship between physical work factors and MSDs. The risk factors for MSDs are presented in Table 2 [14]. 

In the case of Spain, physical demands at work have been identified as the leading cause of work injuries [15]. In addition, the relative risk of musculoskeletal injury has been estimated based on the Continuous Sample of Working Lives obtained from the Social Security Office [16]. Categories of workers at greatest risk in the manufacturing sector are male workers, young workers and low-skilled manual workers. 

However, from the preventive point of view, it is also important to identify the underlying mechanisms in order to design specific actions to fight the causes of musculoskeletal diseases. Although individual characteristics, psychosocial factors and organizational factors are important as well, most preventive systems intervene to control risk factors directly associated with task design and working conditions [17].

In this sense, the investigation of accident and disease investigations is one of the most commonly used tools to identify the causes of MSDs and the barriers to preventing MSDs. In fact, in Spain, as in the rest of Europe, it is mandatory to investigate all MSDs in order to decide if the working conditions are appropriate and to propose additional preventive activities, if necessary, according to the Framework Directive 89/391/EEC [18]. This framework directive includes different processes to be implemented by enterprises regarding MSDs: risk assessment and investigation of causes of the reported MSDs. Furthermore, the Spanish Law implementing the Framework Directive in Spain (Ley 31/1995 de Prevención de Riesgos Laborales) includes as mandatory the investigation of all damage to workers due to occupational exposure, including occupational diseases.

On one hand, risk assessment is a proactive process oriented to identify possible causes and factors of MSD from the preventive point of view so enterprises can adopt preventive measures as a result of risk assessment to reduce the risk of MSD. According to OSHAS 18001, risk assessment could also be defined as the process of evaluating the risk arising from the hazard (combination of the likelihood of a hazardous event or exposure and the severity of injury or ill health that can be caused by the event of exposure).

On the other hand, investigation of reported MSDs is a reactive process oriented to review causes and factors in the MSD reported in order to update risk assessment or enforce the implementation of preventive measures already proposed. According to International Labour Organization, investigation could also be defined as the process used to identify how and why an undesired event (occupational disease or injury) occurred and establish actions required, preventing a similar event [19]. 

Both risk assessment and investigation are a responsibility of the managers, although in Spain this is usually delegated by managers to occupational health and safety specialists in the workplace. In most cases they belong to an accredited occupational health and safety service. In Spain, occupational health and safety specialists have a university degree and at least 650 h of post-graduate university training, according to the syllabus included in Royal Decree 39/1997 [20].

It is remarkable that there are so many technical and scientific documents regarding the investigation of occupational accidents [21], but so few references for the investigation of the causes of diseases. Indeed, no validated references were found for such tasks in main scientific repositories such as Web of Science and Scopus. However, in the last few years, there has been an interest in the development of investigation methods, and it is expected that validated methods for disease investigation could soon be defined [18,22].

The investigation of MSDs reported is oriented to the identification of causes. In this study, a cause is defined according to Rothma [23] (p. 588) as “an act or event or a state of nature which initiates or permits, alone or in conjunction with other causes, a sequence of events resulting in an effect”. In the scope of occupational disease investigations, only primary causes related to working conditions and work organization were analysed, as they are directly connected to the working conditions in the enterprise. The role of secondary causes (latent causes), such as individual characteristics and psychosocial risk factors, was not included in the scope of occupational disease investigations, although much evidence of their importance is available. Individual characteristics such as the previous conditions of the worker, or risky non-work activities can lead to diseases without any cause related to occupational exposure.

Once a possible cause is identified, enterprises need to adopt preventive measures. Unfortunately the scientific evidence of the effectiveness of the preventive measure is not always available, and in most instances no evidence supports the ergonomic interventions proposed [24]. However, there is evidence for one of the most common measures, job rotation, showing that has a positive correlation with higher job satisfaction [25]. According to [26] we define job rotation as workers rotating between tasks within a job or between activities as a means to vary different levels of exposures where tasks are combinations of actions comprising functional operations and activities as the postures and/or movements to perform these tasks. 

### 1.3. Scope and Objectives of This Research

This study is based on survey data provided by workplaces with reported occupational diseases. In most cases, the data are based on investigations performed by safety practitioners as specialists in the workplaces where the diseases were reported. 

This research is based on the results of one of the actions of the First Andalusian Occupational Safety and Health Strategy dedicated to reducing the prevalence of MSDs [27]. This study focuses on an analysis of the investigations performed in workplaces with more than one officially reported disease classified as musculoskeletal. The reason for only analysing workplaces with more than one reported case is to eliminate possible spurious cases. It is important to consider that Andalusia is one of the largest regions of Europe, and represents approximately 15% of the Spanish workforce [28].

The main objective of this study is to analyse the role of mandatory investigation of the causes related to working conditions. According to Framework Directive 89/391/EEC [18], investigation of causes of MSDs reported is an important task within the prevention cycle and should include identification of the risks of the diseases and assessment of the control of those risks. If, as a result of the investigation, further risk assessments or preventive measures are undertaken, the investigation has provided feedback in the prevention cycle, improving safety management. 

Therefore, the purpose of this research is the evaluation of the usefulness of the investigations of occupational musculoskeletal disorders and specifically on the re-assessment of the risk of musculoskeletal disorders and identification of preventive measures. In order to pursue that objective, a set of reported occupational musculoskeletal disease investigations was analysed based on a self-reported questionnaire. Specifically, the role of the investigations in the prevention cycle and in the adoption of preventive measures was studied. 

The secondary objective is the identification of working conditions and corrective actions in the workplaces reporting musculoskeletal diseases. This objective offers a first cross-sectional insight into what corrective actions have been adopted by workplaces as a result of the investigations results during the period studied and was used as part of the evaluation of the First Andalusian Occupational Safety and Health Strategy. 

## 2. Materials and Methods

### 2.1. Occupational Disease Investigation Reports

In this study we included all the diseases which were officially reported in the year 2010 (from 1 June to 31 Dec) and the occupational disease proceedings were finished before 30 April 2011. A report file is said to be finished when the worker has recovered and, if there is a sick leave, returned to work. Thus, diseases reported where the worker was still on sick leave were not included.

Likewise, to be included in this study, the diseases had to be classified as group 2 and within subgroups B to G in the CEPROSS codification system. At the same time, the workplace needed to have had at least two musculoskeletal diseases reported in the year 2010 with the same code. This criterion was used to avoid the inclusion of spurious events. 

According to these criteria, 67 workplaces were included with a total of 168 occupational diseases reported. These workplaces received an official letter inviting them to participate in the research with an enclosed questionnaire approved by the Consejo Andaluz de Prevención de Riesgos Laborales (which is a tripartite participation body with representation of the labour authority, unions and employers’ associations). Consejo Andaluz de Prevención de Riesgos Laborales is the body in charge of the evaluation of actions in the First Andalusian Occupational Safety and Health Strategy, and is in particular responsible for improving occupational health and safety policies, if needed, in light of the results of this action.

**Figure 1:**
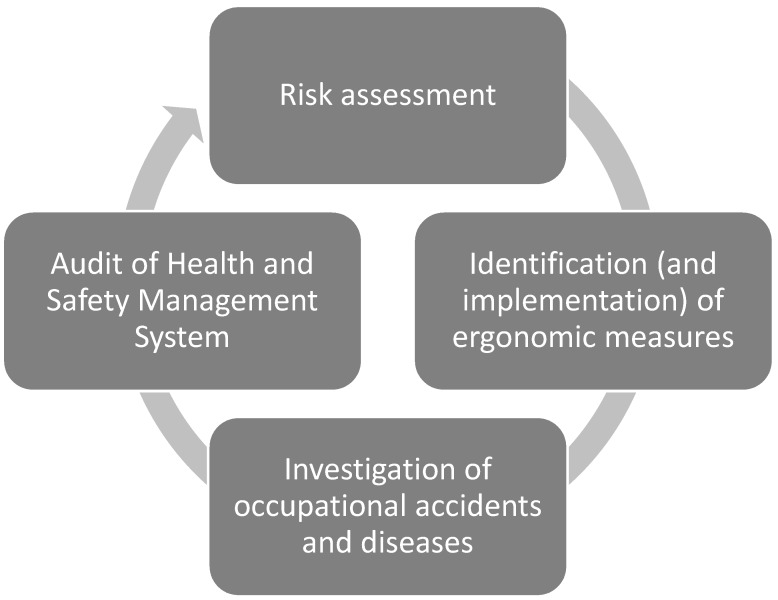
Prevention cycle.

The enclosed questionnaire was designed by the experts of the labour authority in Andalusia. The questionnaire included the enterprise’s main responsibilities regarding occupational disease prevention. The implicit conceptual framework of the questionnaire developed was based on the regulations and was designed with reference to the cycle of continuous improvement presented in Figure 1.

The questionnaire was designed to be completed by an Occupational Health and Safety Specialist in ergonomics at each workplace on behalf of the enterprise. The questionnaire (translated into English) is presented in Table 3. 

From the set of 67 workplaces contacted to answer the questionnaire, 46 questionnaires were received completed (69% of workplaces surveyed) with a total of 114 occupational musculoskeletal diseases investigated in those 46 workplaces (68% of occupational musculoskeletal diseases in the workplaces surveyed). These workplaces employed 15,260 workers.

The questionnaires were reviewed to identify those lacking in quality or containing incomplete information. The criteria used were that questionnaires should have all objective items (1–7 and 11–12, see Table 3) answered and have enough information included in the descriptive items (8–10, see Table 3) to allow the experts of the labour authority to understand the task, risk and measures adopted. 

After quality evaluation of the answers, only 41 questionnaires were considered appropriate for inclusion in the analysis (61% of the workplaces surveyed) with 98 occupational musculoskeletal diseases (58% of occupational musculoskeletal diseases in the workplaces surveyed). These workplaces employed 13,201 workers. In 39 of the 41 of the workplaces an Accredited Health and Safety Service was in charge of ergonomic risks in behalf of the enterprise. 

Regarding economic activities, the most frequent were from “wholesale and retail trade; repair of motor vehicles and motorcycles” with 14 questionnaires, “manufacturing” with 12 questionnaires and “administrative and support service activities” with 7 cases. There is a good answer rate in most of the economic activities except for “water supply; sewerage; waste management and remediation activities” with 12%, and “accommodation and food service activities” with 17% (see Table 4).

Regarding the occupations, the most frequent were “craft and related trades workers” with 9 questionnaires and “elementary occupations” with 8 cases. There is a good answer rate except for “professionals” with 0% and “technicians and associate professionals” with 33% (see Table 5).

Regarding physical activities, the most frequent were vegetable processing workers and supermarket assistants, each with six workplaces, and cleaning operators and meat processing workers, each with five workplaces.

All enterprises performed the investigation of causes, some of them before the survey was received (28 cases) and some of them as a consequence of the survey (13 cases).

### 2.2. Methods

The analysis of the data was performed with descriptive statistics. Most items were yes/no questions. Occupations were classified according to International Standard Classification for Occupations of the International Labour Organization, version 2008 (ISCO-08) at the two-digit level [29]. Diseases reported were classified according to CEPROSS code [9]. Descriptive items were coded by narrative analysis with the following criteria:

- For tasks, a brief description was used.
- For risk factors, as they were descriptive, they were assigned to one of the primary risk factors according to the National Institute for Occupational Safety and Health [9]; see Table 2. This is consistent with Punnet and Wegman [4], who identified repetitive movements, forceful exertions, vibrations and forced or awkward postures as risk factors.
- For corrective measures, the possibilities were identified according to National Occupational Research Agenda for Musculoskeletal Disorders as engineering controls, administrative (organizational) controls and personal protective equipment [30].

Analysis of significant relationships between categorical variables was performed using the Φ coefficient method as described by Chi et al. [31]. The Φ coefficient indicates the association between two categories of variables (e.g., correlations between risks and measure proposed).

## 3. Results

As indicated above, there were 41 questionnaires available for analysis, since 26 questionnaires were not available because they were not answered or were discarded. In order to identify possible bias in both groups, a difference-in-proportion test was developed between these 41 workplaces and the 26 workplaces not included in the research. The result of the test of difference in proportions did not show any significant differences.

The results of dichotomic answers of the survey are presented in Table 6 and Table 7 in order to assess differences whether the enterprise knew that a disease notification has been made or not. Regarding the assessment of occupational risks of MSDs, a quantitative method of evaluation was used in only one of the 41 cases. As shown in Table 7, as a consequence of this action, some of the enterprises performed an investigation before submitting the questionnaire.

The analysis of the implementation and performance of the prevention cycle is presented in Table 8. Note that in this table we do not differentiate if the enterprise knew the existence of a disease notification.

The results of the survey for attributed causes are presented in Table 9. Note that all cases performed the investigation and that one disease notification can have more than one attributed cause.

The proposed preventive measures to eliminate the causes of the reported diseases are presented in Table 10. In 16 cases only one measure was proposed and in 23 cases two measures were proposed. In two cases no measure was proposed. Therefore 62 measures were proposed. 

The information of the associations between measures proposed and each of the risks identified is presented in Table 11. 

Finally, of the 41 cases analysed, in 39 cases (95% of all cases) measures have being planned as a consequence of the risk assessment. However, in only 30 cases (73% of all cases) the preventive measures were implemented because in 9 cases (22% of all cases) although measures have being planned, those measures were no implemented

## 4. Discussion

From the set of 67 workplaces selected with 168 occupational diseases, 46 questionnaires were received completed (69%) with a total of 114 occupational diseases investigated (68%). The proportion of questionnaires with enough quality was high (41 of the 46, 89%).

Within the 41 cases analysed, in 13 cases (32% of the cases analysed) the workplace reported that occupational musculoskeletal diseases were not known to be present among employees when they received the survey, and as a consequence no investigation had been conducted before the survey. Therefore, one of the first effects of the research was to provide information. This can be explained because, in Spain, the communication of occupational diseases from the mutual system to workplace managers is not mandatory, although the information must be available upon request of the workplace, so it depends on the occupational health and safety management of the workplace. 

Regarding the assessment of occupational risks of MSDs, a quantitative method of evaluation was used in only one of the 41 cases, although it is one of the most explored issues regarding musculoskeletal disorders [32,33,34]. In addition, all workplaces surveyed had assessed the occupational risks of musculoskeletal disorders, but in four cases (10% of all cases) the risk assessment failed to identify the attributed risk of the reported disease. Thus, disease investigation enabled the improvement of the prevention cycle, and after the investigation all workplaces had identified the risks of MDSs.

On the other hand, according to the questionnaire, when the risk assessment had previously identified the risk, preventive measures have been planned in all cases. However, when preventive measures were planned, in seven cases (14% of all cases) the planned preventive measures were not implemented yet. Thus, only in 30 cases, the prevention cycle had been completed totally and yet the occupational musculoskeletal disease occurred. Therefore, a completion of the prevention cycle did not succeed in prevention. It must be born in mind that diseases can be multi-causal, and that previous conditions and out of work exposure can offer feasible explanations.

Regarding the attributed causes in the investigations, it was only in one of the cases analysed that the investigation performed by the enterprise did not find any cause. In terms of the attributed causes, the most frequent were repetitive movements (12 cases), repetitive movements combined with load manipulation (12 cases) and load manipulation (five cases). Based on this causes, eight different preventive measures were proposed, all of them well discussed in the literature. Although the most effective preventive measures are those related to elimination of exposure through design or changes in equipment, as opposed to those involving training or work organization [35], the most frequent preventive measures proposed were “job rotation” (22 cases) and “training” (21 cases). In summary, the most frequent cause identified is the “risk of repetitive movements” and the most frequent measure proposed is “job rotation”. In fact, it was found that in eleven of the cases both the “risk of repetitive movements” and “job rotation” were identified. 

It stands out that analysis of accident causes is a useful tool in occupational safety [36], but the analysis of disease causes is not equally developed and thus requires further research. It is also remarkable that no guidelines or technical recommendations exist for occupational disease investigations approved by the Instituto Nacional de Seguridad e Higiene (which is the National Occupational Health and Safety Institute in Spain), whereas for accident investigations [37] many technical references have been published. 

### Limitations and Future Research

This research is based on a self-reported questionnaire. Results rely on the accuracy of the data gathered and have not been double checked. Nevertheless, the purpose of this research is to analyse the usefulness of mandatory investigations, and their role in the prevention cycle, thus at least the results reflect the opinion of the enterprises and their occupational health and safety specialists on the effect of this investigation.

This research is not oriented to the quantification of the prevalence of certain risks or the accuracy of the investigations performed, but rather to identify the role of the investigations in the prevention cycle, the difficulties of the enterprises in complying with this task, and the improvements needed in terms of public policies.

This research has an obvious limitation as to the scope of the research, both temporal and regional. Similar research should be undertaken in other European countries in order to help policy makers adopt new directives to improve the use of disease investigations and also improvements in the notification methods. Nevertheless, the identification of the absence of academic research on occupational disease investigations in comparison with accident investigation is a finding in itself that indicates an important area of research for the future.

Specific analysis of the quality of the investigations needs to be analysed in future research, but first the investigation methods need to be validated.

## 5. Conclusions

All enterprises performed the investigation, some of them (28) before the survey was received, and others (13) as a consequence of the survey. Therefore, this kind of action has an important effect in the promotion of the investigation process, even without further enforcement. This is essentially the case because the notification system is not well designed, and an important number of enterprises are not aware of the diseases reported. Future public strategies with respect to occupational health and safety should include these kinds of interventions.

Disease-cause investigations, similar to accident investigations in the European Framework Directive, can play an important role in the definition of appropriate preventive measures. Of the 41 cases analysed, only in two cases were no measures proposed. A total of 62 measures were proposed as result of the investigations. Therefore, the impact of these disease investigations on the improvement of prevention cycle is quite important.

Thus, the analysis of the cases surveyed in this study indicates that investigation of disease causes can be a useful tool in improving the prevention cycle. 

Occupational health and safety specialists have to perform this mandatory activity with a lack of validated methods for disease investigation. Research is needed to identify and validate investigation methods for occupational diseases such as musculoskeletal disorder. Research of a musculoskeletal nature should be started with the expert validation of the possible causes of musculoskeletal disorders, and with the specification of the causation models. At the same time, the codification of the circumstances of occupational disease is needed to facilitate the analysis and comparability of findings. With that in mind, the European Union should include new variables for the occupational disease notification process.

Regarding the evaluation of investigation of occupational musculoskeletal diseases in Andalusia, this research leads to the following conclusions in the scope of the evaluation of the First Andalusian Occupational Safety and Health Strategy:

- Quantitative methods are not being used to assess musculoskeletal risks.
- Notification to workplaces needs to be improved in order to allow all workplaces to develop and implement an investigation procedure.

## Figures and Tables

**Table 1:** Musculoskeletal diseases reported in Andalusia in 2010.

Code of Diseases (EU)	Subgroup of Disease (CEPROSS)	Description	Number of Cases Reported in 2010
505.01 505.02	2B	Angioneurotic or osteoarticular diseases of the hands and wrists caused by mechanical vibration	13
506.11 506.12 506.13	2C	Pre-patellar and sub-patellar bursitis, olecranon bursitis or shoulder bursitis	14
506.21 506.22 506.23	2D	Diseases due to overstraining of the tendon sheaths, of the peritendineum or of the muscular and tendonous insertions	302
506.40 506.45	2F	Paralysis of the nerves due to pressure or carpal tunnel syndrome	116
506.30	2G	Meniscus lesions following extended periods of work in a kneeling or squatting position	2
Total occupational diseases reported in 2010			447

EU = European Union. MSDs = Musculoskeletal Disorders. CEPROSS = Sistema de Comunicación de Enfermedades Profesionales en la Seguridad Social (Notification System for Occupational Diseases of the Social Security).

**Table 2:** Levels of intervention and risk factors [14].

Level	Risk Factors
Primary	Force, Work posture, Repetition, Contact stress, Duration and magnitude
Secondary	Environmental conditions (cold, vibration, illumination) and Work organization (work recovery cycles, task variability, work rate)

**Table 3:** Questionnaire used in the survey approved by the Consejo Andaluz de Prevención de Riesgos Laborales.

Item	Description	Possible Answers
1	Did you know of the existence of the reported disease?	Yes/No
2	If answer to item 1 is yes, has an investigation been conducted before the survey?	Yes/No
3	Were the occupational risks assessed before the survey?	Yes/No
4	If answer to item 3 is yes, has the risk causing the disease been identified?	Yes/No
5	If answer to item 4 is yes, have preventive activities been planned?	Yes/No
6	If answer to item 5 is yes, have preventive activities been implemented?	Yes/No
7	What was the occupation of the worker?	ISCO code
8	What were the tasks?	Descriptive
9	Which are the risks identified?	Descriptive
10	After the investigation, what corrective measures have been adopted?	Descriptive
11	Was there any medical examination of the worker related to the disease?	Yes/No
12	Disease	CEPROSS code

**Table 4:** Distribution of surveyed cases: economic activity according to NACE-2009.

NACE	Description	Included (Total)
C	Manufacturing	12 (14)
E	Water supply; sewerage; waste management and remediation activities	1 (8)
F	Construction	2 (3)
G	Wholesale and retail trade; repair of motor vehicles and motorcycles	14 (19)
H	Transporting and storage	0 (2)
I	Accommodation and food service activities	1 (6)
N	Administrative and support service activities	7 (9)
O	Public administration and defence; compulsory social security	2 (3)
R	Arts, entertainment and recreation	0 (2)
S	Other services activities	2 (3)

Note: NACE code = Code of economic activity according to European Classification of Economic Activities.

**Table 5:** Distribution of surveyed cases: economic activity according to ISCO-2008.

ISCO	Description	Included (Total)
2	Professionals	0 (3)
3	Technicians and Associate Professionals	2 (6)
5	Services and Sales Workers	3 (5)
6	Skilled Agricultural, Forestry and Fishery Workers	3 (4)
7	Craft and Related Trades Workers	9 (15)
8	Plant and Machine Operators and Assemblers	5 (7)
9	Elementary Occupations	8 (12)
Not applicable		11 (15)

Note: ISCO code = Code of the occupation according to International Standard Classification for Occupations of the International Labour Organization, version 2008.

**Table 6:** Analysis of dichotomic answers when the enterprise knew that the disease notification has been made. Total number of cases: 28.

Item	Description	Yes	% of Cases
2	Has an investigation been conducted before receiving this survey?	23	82
3	Were the occupational risks of the disease assessed before receiving this survey?	28	100
4	If answer to item 2 is yes, has the risk causing the disease been identified?	26	92
5	If answer to item 3 is yes, have preventive activities been planned?	26	92
6	If answer to item 4 is yes, were preventive activities implemented?	21	75

**Table 7:** Analysis of dichotomic answers when the enterprise did not know that the disease notification has been made. Total number of cases: 13.

Item	Description	Yes	% of Cases
2	Has an investigation been conducted before receiving this survey?	5	38
3	Were the occupational risks of the disease assessed before receiving this survey?	13	100
4	If answer to item 2 is yes, has the risk causing the disease been identified?	11	85
5	If answer to item 3 is yes, have preventive activities been planned?	11	85
6	If answer to item 4 is yes, were preventive activities implemented?	9	69

**Table 8:** Prevention cycle performance. These questions are regarding the activities performed before the investigation.

Phase	Number of Cases	% of Cases in Previous Phase	% of All Cases
Were the occupational risks of the disease assessed?	41	-	100
Has the risk causing the disease been identified?	37	90	90
Have preventive activities been planned?	36	97	88
Were preventive activities implemented?	30	83	73

**Table 9:** Causation pattern attributed in the investigation performed.

Causation Pattern	Without Causes	One Cause	Two Causes	Three Causes
No causes attributed	1	0	0	0
Vibration	0	1	0	0
Absence of appropriate PPE	0	1	0	0
Load manipulation	0	5	0	0
Repetitive movements	0	18	0	0
Repetitive movements + forced/awkward postures	0	0	12	0
Repetitive movements + load manipulation	0	0	2	0
Repetitive movements + forced/awkward postures + load manipulation	0	0	0	1
Total	1	25	14	1

Note: PPE = Personal protective equipment.

**Table 10:** Preventive measures proposed as consequence of the investigation.

Preventive Measures Proposed	No. of Cases
Job rotation	22
Training	21
New Equipment	6
PPE	5
Re-design of workstations	2
Re-organization of tasks	2
Improvement in maintenance of equipment	2
Re-assessment of occupational risks	1
Total	62

Notes: PPE = Personal protective equipment. One case can have more than one measure proposed.

**Table 11:** Significant associations between risk identified and measures proposed.

Risk Identified (Number of Cases)	Measure Proposed (Number of Cases)	Φ Coefficient	Number of Cases with the Association of Risk and Measure
Repetitive movements (33)	Job rotation (22)	0.53 **	11
Vibration (1)	Improve maintenance of equipment (1)	0.70 ***	1
Absence of appropriate PPE (1)	Use of PPE (1)	0.42 **	1

** *p* < 0.01; *** *p* < 0.001; PPE = Personal protective equipment.
